# Antimicrobial and Antioxidant Activities of Natural Compounds: Enhance the Safety and Quality of Food

**DOI:** 10.3390/foods9091145

**Published:** 2020-08-20

**Authors:** Maria Leonor Faleiro, Graça Miguel

**Affiliations:** 1 Algarve Biomedical Center, Faculdade de Ciências e Tecnologia, Universidade do Algarve, Campus de Gambelas, 8005-139 Faro, Portugal; 2 Mediterranean Institute for Agriculture, Environment and Development, Faculdade de Ciências e Tecnologia, Universidade do Algarve, Campus de Gambelas, 8005-139 Faro, Portugal; mgmiguel@ualg.pt

Nature has offered us a tremendous diversity of natural compounds, for which antimicrobial and antioxidant properties have been intensively explored and nowadays are plenty recognized. During the last decades both the antimicrobial action of natural compounds (preventing and limiting microbial growth) and their antioxidant properties (reducing the oxidation of fats and limiting the ripening and browning of fruit and vegetables after harvesting) have been intensively investigated, particularly in the food packaging sector, evidencing that they may represent an effective eco-friendly approach to enhance the safety and quality of food products, without an environmentally deleterious impact [1,2,3,4,5].

Food preservation is one of the most interesting applications of several groups of natural products and plant secondary metabolites by virtue of their antimicrobial and antioxidant properties, particularly phenolic compounds (e.g., flavonoids, tannins, floroglucinols), essential oils (e.g., carvacrol, eugenol, thymol, 1,8-cineole), organic acids (e.g., lactic acid, acetic acid), bacteriocins (e.g., nisin, lactocin S, pediocins), lysozymes, lactoferrins [5,6,7,8,9].

There are some challenges on the application of these natural products to food preservation, particularly their degradation and volatilization. To overcome these limitations several approaches have been explored, namely their encapsulation in the form of emulsions, use of cyclodextrins, liposomes, core shell nanofibres [10,11,12,13,14]. The use of these natural products in combination with nanobiotechnology will greatly contribute to food quality and safety.

The main goal of this Special Issue was to evidence the most recent accomplishments on the identification of new natural products, new methods for the determination of both antimicrobial and antioxidant activities, and the incorporation of natural products in matrixes at the nano level to guarantee effective activities and highlight future challenges.

With this perspective Kacániová and colleagues [15] evaluated the antioxidant, antimicrobial and antibiofilm activity of the essential oil (EO) of *Coriander sativum* from Hanus, a.s. (Slovakia). The tested essential oil of *C. sativum* was mainly constituted by β-linalool 66.07%, camphor 8.34%, geranyl acetate 6.91% and cymene 6.35%. The biofilm forming bacteria, *Stenotropomonas maltophilia* and *Bacillus subtilis* were susceptible to this EO and at 0.1% their ability to produce biofilm was impaired as determined by the matrix-assisted laser desorption ionization time-of-flight mass spectrometry (MALDI-TOF MS) Biotyper. The authors also observed that the use of coriander EO prevents the development of the mycelial growth of the fungus *Penicillium expansum* MK-SF 33 on bread (with 41.467 ± 0.881% of moisture and an a_w_ (water activity) value of 0.945 ± 0.002). Regarding the antioxidant activity the coriander EO showed a radical scavenging activity of 51.05%.

Additionally bearing in mind the use of natural compounds to improve food safety, Šimunović et al. [16] explored the action of the EO and the ethanolic extract of culinary herb winter savory (*Satureja montana*) against the foodborne pathogen *Campylobacter jejuni* that is implicated in gastrointestinal diseases and also in a severe neurological disorder denominated by Guillain–Barre syndrome. The concern about the increase in antibiotic resistance developed by foodborne pathogens is rising, and the antibiotic resistance of isolates of *C. jejuni* has been noticed. So, the use of common culinary herbs may constitute an excellent alternative to control the spread of these problematic foodborne pathogens. The *S. montana* EO used in the study of Šimunović [16] showed to be enriched in carvacrol, thymol, thymoquinone, *p*-cymene, and γ-terpinene, also the ethanolic extract showed to be enriched in carvacrol and thymoquinone but also in rosmarinic acid, the main component. The authors tested the activity of the pure main components, EO and the ethanolic extract where the pure components of carvacrol, thymol and thymoquinone demonstrated the highest anti-*Campylobacter* activity, in contrast with the EO and ethanolic extract. Furthermore, combinations of the pure components evidenced high synergistic activity. In this study the authors also explored the ability of the ethanolic extract and the EO to inhibit the efflux pump of *C. jejuni* and cause the disruption of the membrane integrity. Their findings evidenced that the ethanolic extract of *S. montana* is a good efflux pump inhibitor in comparison with the known efflux pump reserpine, however the pure EO components were devoid of efflux pump inhibition activity. Regarding the impact on the disruption of the membrane integrity both EO and ethanolic extract were able to cause disruptive actions on the bacterial membrane, which were concentration dependent. Intriguingly, the pure compounds showed a lack of disruptive activity.

Additionally, the authors observed the role of the CmeGH efflux pump (Cme for *Campylobacter* multidrug efflux) on the resistance of C. jejuni to both S. montana EO and ethanolic extract, in contrast with the CmeABC efflux pump that was only involved in the resistance to the ethanolic extract. Although the resistance effect to the EO and ethanolic extract did not contribute to the development of a higher resistance profile involving increased efflux in the tested C. jejuni strain, this study highlights a crucial aspect on the use of natural products as potential agents of resistance development [16].

Food preservation approaches are essential for controlling spoilage microorganisms and Petruzzi et al. [17] studied the use of Italian propolis to control the growth of several spoiler microorganisms, such as *Pseudomonas putida*, *P. fluorescens*, *Hafnia alvei*, *Enterobacter* spp., *Lactobacillus plantarum*, *Sacharomyces cerevisae* and *Fusarium oxysporum*. The authors used two technical approaches to evaluate the impact of the propolis on the microbial growth: the classical viable count and the determination of the growth index using a low and high level of inoculum taking in consideration modelling functions. The tested propolis sample did not origin a meaningful growth inhibition, instead a delay of the microbial growth was observed, an effect that from the point of view of food preservation is desirable.

Preservation of fruit is challenging due to its structural and physiological characteristics that compromise its long-term storage. The use of edible coatings containing components with antimicrobial and antioxidant proprieties is one of the approaches examined to overcome these limitations. With this perspective Gago et al. [18] evaluated the impact on the storage of ‘Rocha’ pears coated with alginate-based nanoemulsions supplemented with lemongrass essential oil (LG) and citral (Cit). The nanoemulsion treated fruits were stored at 0 °C and at 95% humidity over six months and to mimic the impact on the shelf-life, fruit was placed at 22 °C after two, four and six months. The authors observed a positive impact of coating fruit with nanoemulsions that were able to reduce the fruit colour progression and a better firmness was perceived in comparison with the fruit control. Furthermore, the tested coatings did not disturb the soluble solids content (SSC) and the titratable acidity (TA). The microbial growth evaluated by counting aerobic mesophilic and psychrophilic bacteria and mould and yeasts was lower than the safety limits in all nanoemulsion treatments. The panelists favoured fruit coated with LG 1.25% nanoemulsion. Moreover, fruit treated with LG-nanoemulsions lacked scald symptoms, in contrast with Cit 2% fruit, which showed the highest scald and internal browning symptoms. Since ‘Rocha’ pears show a great storage potential (up to 10 months) under a controlled atmosphere but with risk of developing a chilling injury during long term cold storage, the use of LG nanoemulsion is an encouraging approach to preserve fruit quality and protection from spoilage.

In conclusion, the articles included in the present Special Issue evidence the potential of natural products such as common culinary herbs with antimicrobial and antioxidant properties to be applied in food preservation contributing to maintaining food quality and improving their safety.
